# GCAFlow: Multi-Scale Flow-Based Model with Global Context-Aware Channel Attention for Industrial Anomaly Detection

**DOI:** 10.3390/s25103205

**Published:** 2025-05-20

**Authors:** Lin Liao, Congde Lu, Yujie Gao, Hao Yu, Biao Cai

**Affiliations:** 1 College of Computer Science and Cyber Security, Chengdu University of Technology, Chengdu 610059, China; lllin0831@163.com (L.L.); yujiegao1031@163.com (Y.G.); hyu984799@gmail.com (H.Y.); caibiao@cdut.edu.cn (B.C.); 2 School of Mechanical and Electrical Engineering, Chengdu University of Technology, Chengdu 610059, China

**Keywords:** anomaly detection, unsupervised learning, flow-based models, channel attention mechanism

## Abstract

In anomaly detection tasks, labeled defect data are often scarce. Unsupervised learning leverages only normal samples during training, making it particularly suitable for anomaly detection tasks. Among unsupervised methods, normalizing flow models have shown distinct advantages. They allow precise modeling of data distributions and enable direct computation of sample log-likelihoods. Recent work has largely focused on feature fusion strategies. However, most of the flow-based methods emphasize spatial information while neglecting the critical role of channel-wise features. To address this limitation, we propose GCAFlow, a novel flow-based model enhanced with a global context-aware channel attention mechanism. In addition, we design a hierarchical convolutional subnetwork to improve the probabilistic modeling capacity of the flow-based framework. This subnetwork supports more accurate estimation of data likelihoods and enhances anomaly detection performance. We evaluate GCAFlow on three benchmark anomaly detection datasets, and the results demonstrate that it consistently outperforms existing flow-based models in both accuracy and robustness. In particular, on the VisA dataset, GCAFlow achieves an image-level AUROC of 98.2% and a pixel-level AUROC of 99.0%.

## 1. Introduction

As a fundamental technical methodology, anomaly detection (AD) serves critical roles across diverse fields, with the primary objective of identifying samples that significantly deviate from established normal patterns, including financial fraud detection [1], violence detection [2], medical diagnosis [3], network anomaly detection [4], and industrial defect detection [5]. Within the realm of industrial manufacturing, defect detection, as an essential branch of anomaly detection, is of tremendous significance for increasing product quality, optimizing production processes, and boosting production efficiency. Nevertheless, challenges remain in defect detection, the most prominent of which are the diversity of defect types and the sparsity of data. In real industrial production environments, defects manifest unpredictably with high morphological variability, making it impractical to compile comprehensive datasets encompassing all potential defect categories.

In view of the foregoing issues, recent research has increasingly focusing on unsupervised anomaly detection (UAD) methods. Traditional supervised learning relies on large volumes of labeled defective samples. Alternatively, unsupervised methods require only normal samples for training. They identify anomalous instances by detecting deviations from the learned distribution patterns, effectively performing out-of-distribution (OOD) detection. As a result, unsupervised anomaly detection (UAD) provides significant benefits in industrial defect detection. It reduces dependence on costly annotations and improves generalization to unseen defect types.

In recent years, normalizing flows have been widely used in unsupervised anomaly detection [6,7,8,9,10,11,12]. As a class of generative models with precise probability modeling capabilities, normalizing flows construct bijective transformations from complex data distributions to simple tractable distributions (e.g., Gaussian distributions) via reversible neural networks. Pioneering work by DifferNet [6] integrated multi-resolution image scaling with feature extraction and flow-based density estimation. Similarly, CS-Flow [9] detects image defects by combining a fully convolutional network with a cross-scaled flow, and MSFlow uses fusion flow to exchange multi-scale perceptions. Despite notable progress in feature fusion, these flow-based models typically concentrate on spatial information and neglect the critical channel dimension. We noticed that [13,14] incorporated a channel attention mechanism into their architecture, resulting in improved anomaly detection performance. Building on this insight, we have designed a channel attention module and insert it within our flow model. Concurrently, we modify the model’s subnetwork to bolster its distribution-modeling capabilities. In summary, our contributions are threefold:

- We modify the subnetwork in the flow model to refine its probability estimation by hierarchical convolution and inverted bottleneck.
- We propose a global context-aware (GCA) channel attention mechanism and insert it into the flow-based model to optimize anomaly detection and localization.
- We conduct extensive experiments and GCAFlow performs well on the MvTeC AD, VisA, and BTAD benchmark datasets.

## 2. Related Work

### 2.1. Unsupervised Anomaly Detection

Existing approaches to unsupervised anomaly detection can be broadly categorized into three groups: reconstruction-based methods, embedding similarity-based methods, and flow-based methods.

Reconstruction-based anomaly detection methods rely on models trained exclusively on normal samples to accurately reconstruct normal regions. In contrast, poor reconstruction in abnormal regions leads to significantly higher reconstruction errors, which serve as indicators of anomalies. Most methods use generative models (e.g., auto-encoders [15], variational encoders [16], GANs [17], and diffusion models [18]) to predict anomalies and localize anomalous regions. VAE is trained by maximizing the evidence lower bound (ELBO) while optimizing the reconstruction error and KL divergence. However, this lower bound can be quite slack, leading to suboptimal modeling of complex data distributions. GAN-based methods learn the distribution of normal data through adversarial training, in which a generator produces synthetic normal samples, and anomalies are detected by reconstruction errors. Nevertheless, these methods are prone to mode collapse during training, which limits their robustness. Recently, diffusion models [19,20] have emerged as a powerful tool for anomaly detection tasks. RealNet [21] achieves efficient encoding and reconstruction of normal data by combining a key component, Strength-controllable Diffusion Anomaly Synthesis (SDAS), with other key components, and distinguishing reconstruction errors from anomalies.

Similarity-based embedding methods [22,23,24,25] aim to identify anomalies by learning low-dimensional representations of normal samples and quantifying deviations in the embedding space. PaDiM [24] and PatchCore [25] use CNNs pre-trained on ImageNet [26] to extract the embedded representation of a local patch in an image. The former measures the degree of abnormality by fitting a multivariate Gaussian distribution and computing the Mahalanobis distance, whereas the latter uses k-nearest-neighbor search to quantify the distance between patches in the embedding space and utilize this as an anomaly score. However, as the local patch approach may overlook the global context information of the image, it is prone to misinterpretation for images with complicated structures or variable backgrounds. In addition, embedding methods exhibit heightened sensitivity to noise and sample distribution imbalance, potentially compromising the stability and generalization of anomaly scoring mechanisms.

### 2.2. Normalizing Flows

Normalizing flows [27] are a class of generative models based on invertible transformations. They map complex data distributions to simple prior distributions (e.g., Gaussian) through a sequence of learnable bijective mappings. Superficially, the transformation process in normalizing flows may appear similar to that of diffusion models. Both aim to transform complex data distributions into simpler ones. However, diffusion models [28,29] rely on a stochastic process. They gradually add noise to data during the forward process and generate samples through a learned reverse denoising procedure. In contrast, normalizing flows use deterministic and bijective transformations. While diffusion models often achieve impressive sample quality, they typically require hundreds of denoising steps during generation. Normalizing flows, on the other hand, support tractable likelihood estimation, which serves as a valuable probabilistic measure for anomaly detection tasks. In an unsupervised learning context, normalizing flows can effectively identify anomalous samples by learning the probability distribution of normal data, making them particularly well suited for unsupervised anomaly detection tasks. In order to achieve the bijective transformation (i.e., invertibility) of normalizing flows, researchers have made various attempts. Notably, the design of the affine coupling layers [30,31] has been instrumental in ensuring both invertibility and computational efficiency. Furthermore, Glow [32] extends the capability of normalizing flows by using invertible 1 × 1 convolutions to generalize arbitrary channel-wise permutations.

### 2.3. Flow-Based Methods for UAD

Normalizing flows have attracted growing interest for anomaly detection and our method is developed within this framework.

DifferNet [6] pioneered an anomaly detection approach that integrates feature extractors with normalizing flows. The model resizes input images to three different scales, extracts features using a feature extractor, and then feeds these features to the normalizing flow to estimate their likelihood.

CFLOW [8], grounded in the conditional normalizing flow framework, collaborates with a multi-scale generative decoder to explicitly estimate the likelihood of encoded features. Additionally, it introduces spatial prior information through positional encodings, enhancing the model’s ability to localize anomalous regions.

CS-Flow [9] advances the field by integrating fully convolutional networks with cross-scale normalizing flows. It constructs a multi-scale feature pyramid to extract hierarchical image features and applies cross-scale flows to model inter-scale relationships. By processing feature maps at multiple resolutions in parallel, CS-Flow effectively captures global structures along with fine-grained details, yielding precise likelihood estimation.

Despite its effectiveness, CS-Flow’s cross-scale information interaction demands significant computational resources and memory. To alleviate this problem, MSFlow [10] proposes asymmetric parallel flows and more lightweight fusion flow modules, achieving more efficient information exchange.

### 2.4. Attention Mechanisms

The core idea of the channel attention mechanism is to allow the model to dynamically emphasize different channels of the input, rather than treating all channels equally through uniform averaging. By integrating the channel attention mechanism, models adaptively assign varying weights to input features based on their significance. This approach improves the focus on task-relevant information and boosts both overall performance and generalization ability.

The SE Block [33] extracts global channel information through global average pooling and uses a fully connected two-layer network to generate channel attention weights. These weights are then applied to reweight the input features, enhancing the representational capacity of the model across different channels.

The CBAM Block [34] extends the SE Block by incorporating spatial attention. It first extracts channel attention using global average pooling and max pooling, and then computes spatial attention via convolution. This dual attention mechanism enhances the feature information along both the channel and spatial dimensions.

GCNet [35] proposes a more efficient channel attention mechanism by simplifying NLNet [36], which models global context and captures long-range dependencies through spatial attention. Furthermore, by integrating the SE Block, GCNet optimizes feature recalibration, resulting in better performance in tasks such as object detection and segmentation while preserving computational efficiency.

The ECA layer [37] refines the SE Block by removing fully connected layers and adopting 1D convolution to capture local cross-channel interactions. This modification reduces both parameter complexity and computational overhead while preserving the model’s ability to adaptively recalibrate channel-wise feature responses, leading to a more efficient and effective attention mechanism.

## 3. Methods

### 3.1. Theoretical Background of Normalizing Flows

Normalizing flows are a class of probabilistic generative models based on reversible transformations. They employ the change of variables theorem to transform complex data distributions into simple prior distributions through a sequence of invertible mappings. Specifically, given a complex random variable $x=p_{X}\left(x\right)$ and a simpler latent variable $z=p_{Z}\left(z\right)$, the data density can be written as

(1) $$\begin{array}{cc} p_{X}\left(x\right) & =p_{Z}\left(z\right)\left|\det\left(\frac{\partial z}{\partial x}\right)\right| \end{array}$$

where $\frac{\partial z}{\partial x}$ denotes the Jacobi matrix of the transformation, which describes the local scale changes during the transformation from one distribution to the other. The absolute value of the determinant, $\det|\left(\frac{\partial z}{\partial x}\right)|$, denotes the absolute volume change under this mapping. The log-likelihood of image features can be estimated from $p_{Z}\left(z\right)$ by

(2) $$\begin{array}{cc} \log p_{X}\left(x\right) & =\log p_{Z}\left(z\right)+\log\left|\det\left(\frac{\partial z}{\partial x}\right)\right| \end{array}$$

**Figure 1:**
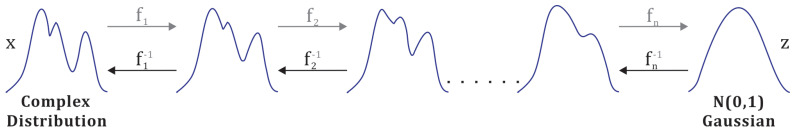
The flow model maps a complex distribution to a simple distribution. *f* is an invertible function, and $f^{-1}$ represents its inverse function.

Model training typically minimizes the negative log-likelihood. Accordingly, maximizing the data log-likelihood $\log p_{X}\left(x\right)$ can be formulated as

(3) $$\begin{array}{cc} \max p_{X}\left(x\right) & \Leftrightarrow \min-\log p_{Z}\left(z\right)-\log\left|\det J_{f^{-1}}\left(x\right)\right|. \end{array}$$

(4) $$\begin{array}{cc} \det J_{f^{-1}}\left(x\right) & =det\frac{\partial z}{\partial x} \end{array}$$

where $J_{f^{-1}}\left(x\right)$ represents the Jacobian of the reversed normalizing flow. In practical applications, a single invertible transformation is usually insufficient; hence, the flow model must be implemented by stacking numerous layers of invertible transformations. Figure 1 illustrates the process of this cascade transformation. The formula can be expressed as below (5).

(5) $$\begin{array}{c} \begin{array}{cc} x & =f\left(z\right)=f_{K}\circ f_{K-1}\circ \ldots \circ f_{1}\left(z\right)\\ z & =f^{-1}\left(x\right)=f_{1}^{-1}\circ f_{2}^{-1}\circ \ldots \circ f_{K}^{-1}\left(x\right) \end{array} \end{array}$$

As illustrated, ensuring the reversibility of the flow model is crucial. A widely adopted solution is the affine coupling layer [30,31], which maintains invertibility and enables efficient computation of the Jacobian determinant. Input variables *x* are split along the channel dimension, and an affine transformation is applied to only one subset of channels. This design maintains the invertibility of the overall mapping. The calculation process for this transformation is as follows:

(6) $$\begin{array}{cc} x_{1},x_{2} & =\mathrm{split}(x) \end{array}$$

(7) $$\begin{array}{cc} x^{\prime } & =x_{2}⊙\exp\left(s\left(x_{1}\right)\right)+t\left(x_{1}\right) \end{array}$$

(8) $$\begin{array}{cc} x^{\prime \prime } & =x_{2}⊙\exp\left(s\left(x^{\prime }\right)\right)+t\left(x^{\prime }\right) \end{array}$$

(9) $$\begin{array}{cc} y & =\mathrm{concat}(x^{\prime },x^{\prime \prime }) \end{array}$$

where s(·), t(·) are affine parameters, which are implemented by subnet.

### 3.2. Overview

**Figure 2:**
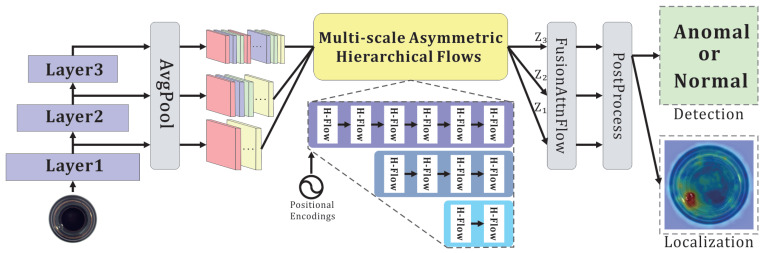
Anomaly detection pipeline of GCAFlow. The subnetwork in the H-Flow adopts hierarchical convolution. In FusionAttnFlow, in addition to performing feature fusion, we incorporate the global context-aware (GCA) module to extract channel-wise attention. PostProcess includes different aggregation strategies for anomaly detection and anomaly localization to generate high-quality anomaly maps.

The detailed structure of GCAFlow is shown in Figure 2. Each input image is processed by the feature extractor to generate three sets of feature maps. After being 2× downsampled by average pooling, the three feature maps are separately fed into the Multi-scale Asymmetric Hierarchical Flows module. This module encodes their intrinsic properties and consists of different numbers of stacked H-Flow blocks in each branch. Moreover, to compensate for the inability of hierarchical convolution to capture the absolute positions of feature vectors, each H-Flow block incorporates positional encoding as a conditional input. Next, FusionAttnFlow deeply fuses the features while efficiently integrating channel information via the global context-aware (GCA) mechanism. Finally, the model separately processes them based on the requirements of detection and localization tasks, producing the final anomaly detection map.

### 3.3. Feature Extractor

Similar to the prevailing normalizing flow methods, GCAFlow requires a single pass through a feature extractor. In our implementation, we adopt the pre-trained WideResNet-50 [38] as a multi-scale feature extractor. Although its final layer captures high-level semantics, exclusive reliance on that output risks losing important textural and structural details. To address this, we extract features from the first three layers. These three sets of features are subsequently used as inputs for GCAFlow training in a sequential manner.

### 3.4. Hierarchical Convolutional Subnetwork

The architecture of the affine coupling layer ensures the invertibility of normalizing flow models. Consequently, designing the subnetworks within the affine coupling layer to improve their feature extraction capability and enable a more effective transformation from complex distributions to simple ones is a critical challenge. Previous flow models [7,8,9,10,39] have focused on optimizing overall architectures but continue to rely on stacks of 1 × 1 or 3 × 3 convolutions in their subnetworks, which limits their distribution-modeling capability.

**Figure 3:**
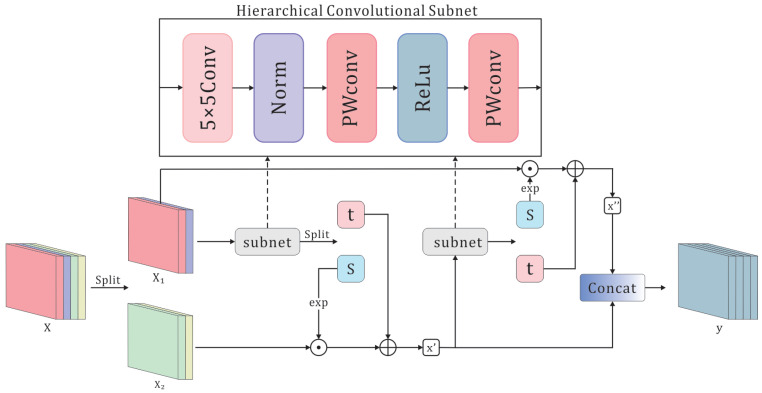
The detailed structure of an H-Flow, where Norm uses layernorm, and PWconv is a 1 × 1 convolution.

To overcome this limitation, we introduce a novel hierarchical flow (H-Flow), as illustrated in Figure 3. It still adopts the affine coupling layer; however, its subnetwork is implemented using a hierarchical convolutional subnetwork.

In a hierarchical convolutional subnetwork, the 5 × 5 convolutional kernel is employed for spatial feature extraction due to its ability to capture a wider range of local information. Owing to its extended receptive field, this kernel can detect texture details and local patterns at elevated levels. Instead of batch normalization, which relies on batch statistics, we use layer normalization (LayerNorm) to normalize channel activations for each sample independently. LayerNorm stabilizes neuron input distributions, mitigates gradient vanishing and explosion, and accelerates convergence. Moreover, by normalizing the scale variations introduced by 5 × 5-kernel outputs, LayerNorm reduces sensitivity to distributional shifts and further enhances training stability and efficiency. Drawing inspiration from [40,41,42], we adopt an inverted bottleneck structure. In this design, the initial 1 × 1 convolution facilitates cross-channel information exchange and expands the channel dimension, allowing the flow-based model to acquire higher-dimensional features. Subsequently, a 1 × 1 convolution restores the channel count to match the input, thereby preserving the reversibility of the flow model.

This hierarchical structure extracts fine-grained spatial details and enables channel-wise feature transformations, thereby bolstering the model’s distribution-modeling capacity. Furthermore, we conducted experiments in Section 4.5 to evaluate the impact of various convolutional kernel sizes and different channel expansion factors on the overall model performance.

### 3.5. Global Context-Aware Module

ECA [37] experimentally proved that the SE Block [33] can damage information integrity by first reducing the dimensionality and then enhancing it when extracting the channel information. Instead, utilizing 1D convolution not only alleviates this issue but also substantially increases computational efficiency. Motivated by this finding, we propose an improved channel attention module that efficiently integrates channel information without sacrificing the integrity of global feature representations.

**Figure 4:**
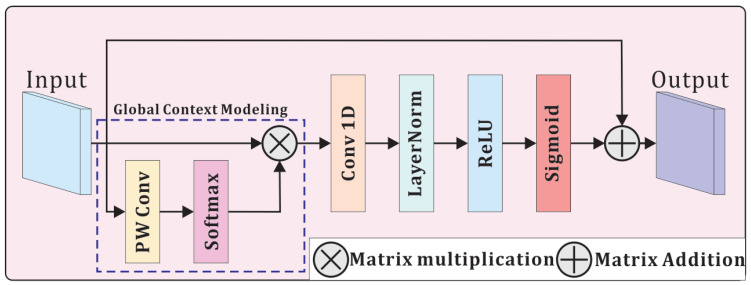
The structure of the GCA module, where PWconv represents 1 × 1 convolution, also known as point-wise convolution.

The proposed module first facilitates cross-channel information interaction via a 1 × 1 convolution, flexibly modeling cross-channel dependencies. Then, a softmax function is applied to compute global spatial attention weights, which are then multiplied with the input via matrix multiplication to extract enriched global channel information. Subsequently, a 1D convolution followed by a sigmoid activation generates the final channel attention weights. Finally, these weights are multiplied with the original input, yielding global semantic information while effectively filtering out redundant features. Figure 4 illustrates the workflow of the GCA module. According to [37], for a channel dimension of *C*, the kernel size *k* of the convolution 1D should be adaptive, giving it the flexibility to capture different ranges of channel dependencies. This kernel size is computed through the formula

(10) $$k=\psi \left(C\right)={\left|\frac{\log_{2}\left(C\right)}{\gamma }+\frac{b}{\gamma }\right|}_{\mathrm{odd}}$$

where ${\left|t\right|}_{odd}$ indicates the closest odd number. We set γ to 2 and *b* to 1. We embed the GCA module into FusionAttnFlow (Section 3.6) to improve the model’s ability to extract contextual information.

### 3.6. FusionAttnFlow

**Figure 5:**
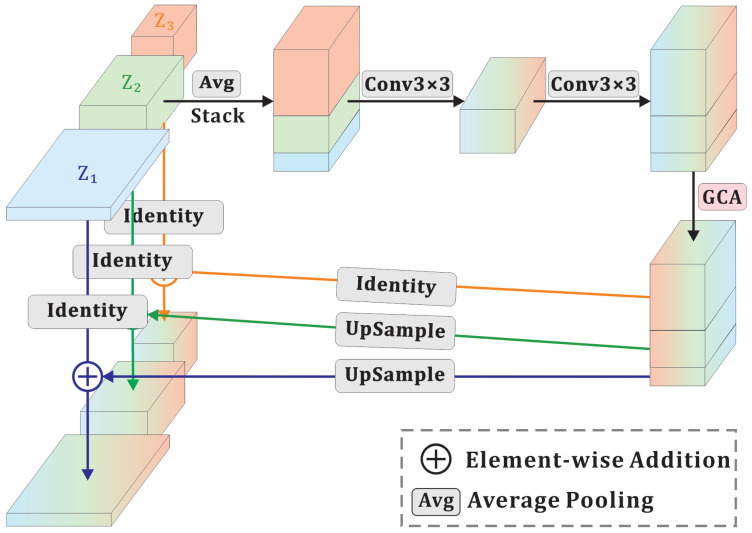
The structure of FusionAttnFlow, where identity represents a no-op transformation that preserves the input.

Fusion flow [10] has been shown to effectively integrate multi-scale information, promoting efficient information sharing. It retains the affine coupling layer structure introduced in RealNVP [31] while receiving inputs from three different scales. These multi-scale features are then integrated using a fusion strategy inspired by HRNet [43]. On this basis, we feed the fused features into the GCA module to extract channel attention. Ultimately, it outputs three feature maps that retain their original shapes. Figure 5 illustrates the architecture of FusionAttnFlow.

The three inputs are maps that are first downsampled to a unified spatial resolution using average pooling (AP). These downsampled maps are concatenated to capture the local information through the 3 × 3 convolution in the bottleneck structure. Given that feature maps at different scales contribute unequally to the final anomaly map, it is both reasonable and effective to assign adaptive weights to different channels. To this end, we employ the GCA module to extract channel weights. Finally, the fused feature map is divided into three segments along the channel dimension. Each segment is upsampled to its original resolution via bilinear interpolation and then combined through element-wise addition to produce the final fused output.

### 3.7. Anomaly Score Generation

The flow framework employs multiple flow blocks to map the true data distribution—defined as the distribution of the dataset—onto a standard normal distribution. The negative log-likelihood (NLL) function output of this process can be used as a defect score map. During the training process, the NLL function for normal samples should tend towards or equal zero, whereas for abnormal samples it should deviate from zero. This is because abnormal samples do not conform to the distribution characteristics of normal samples, resulting in a departure from a zero NLL function. Through this approach, we can filter out samples that do not conform to the distribution of normal samples, thus obtaining an anomaly score map for abnormal samples.

During the anomaly score production phase, the three multi-scale likelihood maps are initially upsampled to match the dimensions of the input image and subsequently merged through element-wise addition to obtain the probability map. According to [10], leveraging multiplicative computation for image-level anomaly detection more effectively mitigates noise, while additive aggregation for pixel-level anomaly localization facilitates the detection of anomalies at various scales. Therefore, the final computation formula is as follows:

(11) $$\begin{array}{cc} P^{\mathrm{sum}} & =\sum_{i=1}^{3}P_{i} \end{array}$$

(12) $$\begin{array}{cc} P^{\mathrm{mul}} & =\prod_{i=1}^{3}P_{i} \end{array}$$

(13) $$\begin{array}{cc} S_{p} & =\max\left(P_{sum}\right)-P_{sum}; \end{array}$$

(14) $$\begin{array}{cc} s_{i} & =\frac{1}{K}\sum_{1}^{K}\mathrm{top}K\left(\max\left(P_{mul}\right)-P_{mul}\right), \end{array}$$

$S_{p},S_{i}$ represents the final image-level and pixel-level anomaly scores. top*K* denotes the largest K values; it is robust against both noise and small anomaly regions.

## 4. Experiments

### 4.1. Dataset

MvTec AD (anomaly detection) [5] is a widely used benchmark dataset for unsupervised industrial anomaly detection. It comprises more than 15 types of industrial products, categorized into object categories and five texture categories. The training set consists of 3629 defect-free images, whereas the test set includes 1725 images, encompassing both faulty and defect-free samples.

Current experimental results on the MvTec AD indicate that the AUROC metric has nearly reached saturation. To further advance the field of anomaly detection, the VisA dataset [44] was introduced in 2022. This dataset also adopts a self-supervised learning approach, encompassing 12 distinct categories and comprising a total of 10,821 images, including 9621 normal samples and 1200 anomalous samples.

To evaluate the generalizability of our method, we further conducted experiments on the BeanTech Anomaly Detection (BTAD) dataset [45], which consists of 2830 images collected from three types of industrial products.

### 4.2. Evaluation Indicators

In anomaly detection tasks, the AUROC (area under the receiver operating characteristic curve) is widely used as an evaluation metric. In this study, we calculate both the image-level AUROC (I-AUROC) and the pixel-level AUROC (P-AUROC) to assess the performance of anomaly detection and the accuracy of anomaly localization. For each dataset, the overall performance of the model is comprehensively evaluated by computing the average image-level AUROC and the average pixel-level AUROC across all categories.

### 4.3. Implementation Details

We begin by resizing the input images to 512 × 512 and then use WideResNet-50 [38] pre-trained on ImageNet-1K [26] to extract multi-level features. To boost computational efficiency, we downsample the features via average pooling and process the resulting feature maps with three multi-scale asymmetric normalization flows. We also incorporate 2D positional encodings as conditional information into all flow blocks and employ a FusionAttnFlow after the final block to enable cross-scale information exchange. Unless otherwise specified, our training strategy follows a one-class approach, where the model is trained exclusively on data from a single class, closely resembling industrial production scenarios. During training, we adopt the Adam optimizer with an initial learning rate of 1 × 10^−4^ and train for 100 epochs with a batch size of 16. All experiments were run on an NVIDIA GeForce RTX 4090 GPU.

### 4.4. Comparisons with Other Methods

In the experiments, we compared GCAFlow with state-of-the-art anomaly detection methods on the MvTec AD, VisA, and BTAD datasets.

The experimental results on the VisA dataset, as shown in Table 1 and Table 2, demonstrate that the proposed method achieves state-of-the-art performance in both image-level anomaly detection and pixel-level anomaly localization tasks. Specifically, in the image-level anomaly detection task, the proposed method attains an average AUROC of 98.2%, significantly surpassing all existing approaches, including the flow-based models MSFlow (97.7%) and Fastflow (82.2%). Similarly, in the pixel-level anomaly detection task, the proposed method achieves the highest average AUROC of 99.0%, outperforming the current best methods, PathCore (98.8%) and MSFlow (98.8%). These results indicate that, compared to existing flow-based approaches, the proposed method exhibits superior accuracy and robustness, and achieves the overall best performance among all compared methods, providing a more advanced solution for anomaly detection tasks.

Table 3 and Table 4 report the performance of our method on image-level anomaly detection and pixel-level localization tasks on the MvTec AD dataset, showing superior results across multiple categories and demonstrating strong generalization and anomaly detection capabilities. In terms of image-level AUROC results, our approach achieves state-of-the-art performance in several categories (e.g., Carpet, Leather, Tile) and attains a total average score of 99.7%, either matching or slightly surpassing the best-performing methods. For pixel-level AUROC evaluation, our method also achieves the best results in multiple categories (e.g., Carpet, Leather, Capsule) and reaches a total average score of 98.4%, approaching the highest reported performance (98.8%). Compared to existing approaches, our method demonstrates competitive results in categories with complex backgrounds (e.g., Cable, Screw) while maintaining overall robustness. In summary, the detection performance on the VisA dataset is inferior to that on MvTec, primarily because the defect regions in the VisA dataset are relatively small, making the detection task more challenging.

Our method also exhibits impressive performance on the BTAD dataset. As shown in Table 5, it ranks first among the listed methods in terms of both average image-level AUROC and average pixel-level AUROC. Remarkably, GCAFlow achieves high accuracy after training for only 10 epochs, underscoring its strong potential for anomaly detection tasks.

**Figure 6:**
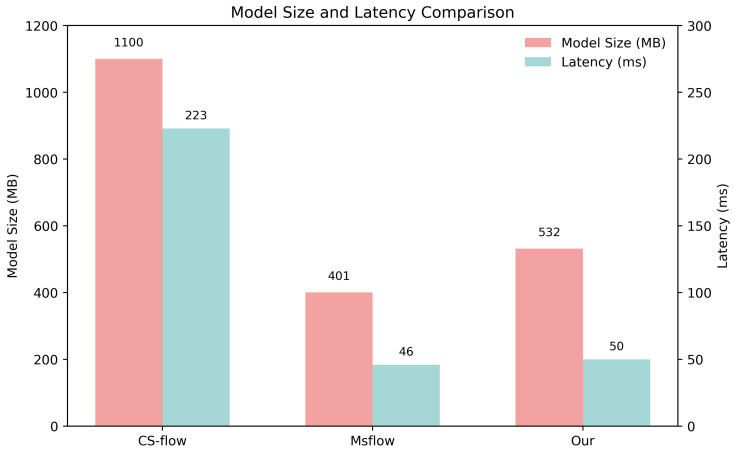
Comparison of model size and inference latency.

We compared the model size and inference latency of two flow-based models with high accuracy. As can be seen from Figure 6, GCAFlow is significantly smaller (51.6%) in terms of model size compared to CS-flow (1100 MB). Although its model size is slightly larger (32.7%) than MSFlow (401 MB), its inference latency is almost the same as MSFlow, with a difference of only 4 ms. Considering the excellent performance of GCAFlow in anomaly detection tasks, it achieves a good balance between accuracy and model efficiency.

### 4.5. Ablation Study

To comprehensively evaluate the detection performance of our model, we conducted extensive ablation studies on the VisA dataset to investigate the impact of key design components.

**The Effect of Different Kernel Sizes in Hierarchical Convolutional Subnetwork.** In our subnetwork architecture, the first convolutional layer is typically designed with a larger kernel to expand the receptive field and capture more comprehensive spatial context information. In this experiment, we conducted a comparative analysis on the impact of different kernel sizes, with the experimental results summarized in Table 6. The results indicate that a 3 × 3 convolution kernel performs exceptionally well for image-level anomaly detection. This can be attributed to the small and often indistinguishable nature of the defects, where local features are more informative than global context. Consequently, a smaller kernel is more effective at capturing the key features necessary to enhance overall detection performance. On the other hand, for pixel-level localization, both 5 × 5 and 7 × 7 convolution kernels yield scores approaching 99, as their larger receptive fields enable the integration of broader contextual information at each pixel. This broader context facilitates discrimination between subtle anomalies and background noise, thereby achieving precise localization. Taken together, a 5 × 5 convolution kernel appears to offer an optimal trade-off, delivering strong performance in both image-level detection and pixel-level localization. **Effect of Varying Channel Expansion Factors in Hierarchical Convolutional Subnetwork.** In this study, we analyze how varying the channel expansion multiplier within the subnetworks affects the flow model’s ability to map features. To achieve this, experiments were conducted using different expansion factors, denoted as α. Table 7 reveals that the model attains its best detection performance when α = 4, suggesting that an appropriately tuned channel expansion can improve the model’s anomaly detection performance.

**Impact of Different Attention Mechanisms in FusionAttnFlow.** To investigate the impact of different attention mechanisms on the distribution modeling capability of flow-based models, we substituted the GCA module in the FusionAttnFlow structure with the SE Block and ECA layer and conducted experiments. The experimental results, reported in Table 8, show that the GCA module significantly enhances the model’s anomaly detection performance, outperforming the other attention mechanisms.

### 4.6. Visualization Results

**Figure 7:**
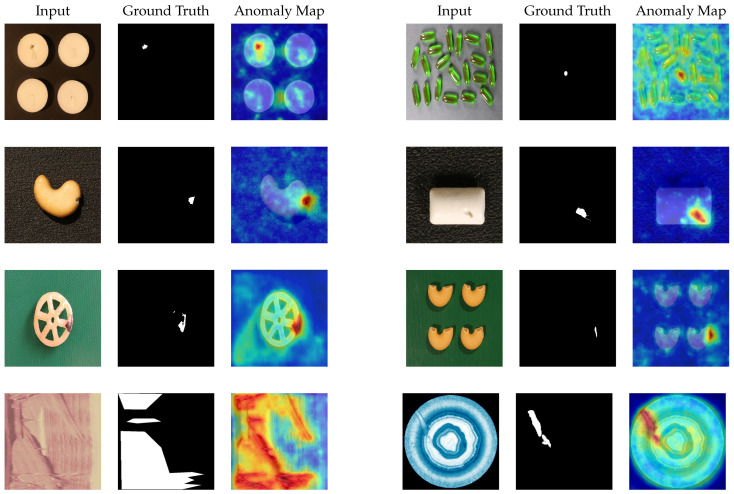
Visualization results of anomaly localization on the VisA and BTAD datasets. The top three rows display the results on the VisA dataset, and the bottom row displays the results on the BTAD dataset.

**Figure 8:**
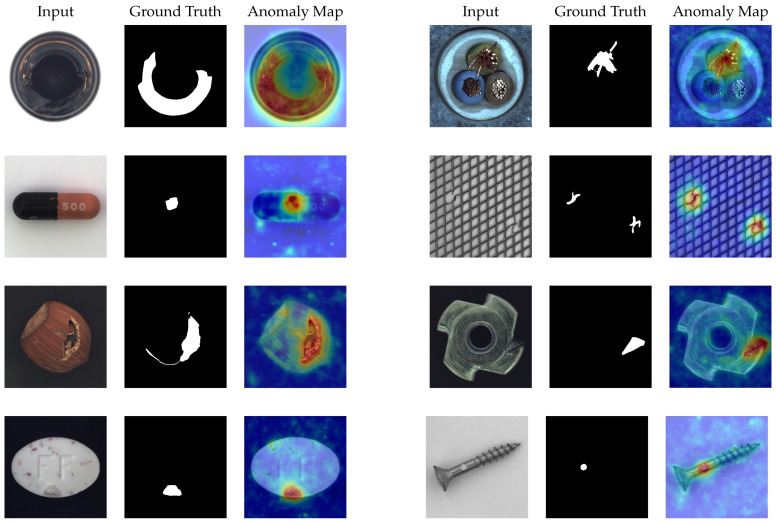
Visualization results of anomaly localization on the MvTec AD dataset.

We visualize the anomaly localization by overlaying the final output of GCAFlow’s anomaly score map with the original image. Figure 7 and Figure 8 show the visualization results of GCAFlow on the MvTec AD, VisA, and BTAD datasets.

## 5. Discussion and Conclusions

Benefiting from the powerful distribution mapping capability of normalizing flows, the method has demonstrated exceptional performance in anomaly detection tasks. To address the limitation of existing flow-based frameworks that primarily focus on spatial information while neglecting channel-wise features, we propose GCAFlow, a novel module that incorporates global context-aware channel attention. Furthermore, to enhance the probabilistic modeling capability of the flow-based framework, we design a hierarchical convolutional subnetwork. Ablation studies indicate that the channel attention module significantly improves model performance. Extensive experiments on the VisA, MvTec AD, and BTAD datasets fully validate the robustness and high accuracy of GCAFlow in industrial anomaly detection scenarios.

However, flow-based models usually need to train a separate model for each category in practical applications, due to the fact that the distributional differences between different categories can interfere with model training. Therefore, in future work we would like to explore a flow-based model that can handle all categories uniformly, thus further promoting the development and application in the field of anomaly detection.

## Figures and Tables

**Table 1:** Image-level AUROC (%) for various anomaly detection methods on VisA. The best results are in bold, and the second-best results are underlined.

Method	SPADE [22]	DRAEM [46]	PathCore [25]	RealNet [21]	Fastflow [39]	MSFlow [10]	Ours
candle	91.0	91.8	**98.6**	96.1	92.8	98.3	98.4
capusle	61.4	74.7	81.6	93.2	71.2	96	**96.8**
cashew	97.8	95.1	97.3	97.8	91.0	**98.6**	98.2
chewinggum	85.8	94.8	99.1	**99.9**	91.4	99.6	99.4
fryum	88.6	97.4	96.2	97.1	88.6	**99.6**	**99.6**
macaroni1	95.2	97.2	97.5	**99.8**	98.3	97.6	98.2
macaroni2	87.9	85.0	78.1	**95.2**	86.3	89.4	92.3
pcb1	72.1	47.6	98.5	98.5	77.4	**99**	98.9
pcb2	50.7	89.8	97.3	97.6	61.9	97.8	**98.5**
pcb3	90.5	92.0	97.9	99.1	74.3	**99.8**	99
pcb4	83.1	98.6	99.6	99.7	80.9	98.2	**99.8**
pipe_fryum	81.1	**100**	99.8	99.9	72.0	98.8	99.5
Average	82.1	88.7	95.1	97.8	82.2	97.7	**98.2**

**Table 2:** Pixel-level AUROC (%) for various anomaly detection methods on VisA. The best results are in bold, and the second-best results are underlined.

Method	SPADE [22]	DRAEM [46]	PathCore [25]	RealNet [21]	Fastflow [39]	MSFlow [10]	Ours
candle	97.9	96.6	**99.5**	99.1	94.9	99.4	99.4
capusle	60.7	98.5	99.5	98.7	75.3	**99.7**	**99.7**
cashew	86.4	83.5	98.9	98.3	91.4	**99.1**	98.8
chewinggum	98.6	96.8	99.1	**99.8**	98.6	99.4	99.3
fryum	96.7	87.2	93.8	96.2	**97.3**	92.7	94.5
macaroni1	96.2	**99.9**	99.8	**99.9**	97.3	99.8	99.8
macaroni2	87.5	99.2	99.1	**99.6**	89.2	**99.6**	**99.6**
pcb1	66.9	88.7	**99.9**	99.7	75.2	99.8	99.8
pcb2	71.1	91.3	99.0	98.0	67.3	**99.2**	**99.2**
pcb3	95.1	98.0	99.2	98.8	94.8	99.3	**99.4**
pcb4	89.0	96.8	98.6	98.6	89.9	98.5	**99.1**
pipe_fryum	81.8	85.8	99.1	**99.2**	87.3	99.1	**99.2**
Average	85.6	93.5	98.8	98.8	88.2	98.8	**99.0**

**Table 3:** Image-level AUROC (%) for various anomaly detection methods on MvTec AD. The best results are in bold, and the second-best results are underlined.

Method	DRAEM [46]	SSPCAB [47]	RD4AD [48]	PatchCore [25]	Simplenet [49]	CS-Flow [9]	MSFlow [10]	Ours
Carpet	97.0	98.2	98.9	98.7	99.7	**100**	**100**	**100**
Grid	99.9	**100**	**100**	98.2	99.7	99.0	99.8	99.8
Leather	**100**	**100**	**100**	**100**	**100**	**100**	**100**	**100**
Tile	99.6	**100**	99.3	98.7	99.8	**100**	**100**	**100**
Wood	99.1	99.5	99.2	99.2	**100**	**100**	**100**	99.8
Average.textuer	99.1	99.5	99.5	99.0	99.8	99.8	**100**	99.9
Bottle	99.2	98.4	**100**	**100**	**100**	99.8	**100**	**100**
Cable	91.8	96.9	95.0	99.5	**99.9**	99.1	99.5	99.6
Capsule	98.5	**99.3**	96.3	98.1	97.7	97.1	99.2	**99.3**
Hazelnt	**100**	**100**	99.9	**100**	**100**	99.6	**100**	**100**
Metal_Nut	98.7	**100**	**100**	**100**	**100**	99.1	**100**	**100**
Pill	98.9	**99.8**	96.6	96.6	99.0	98.6	99.6	99.4
Screw	93.9	97.9	97.0	98.1	**98.2**	97.6	97.8	97.9
Toothbrush	**100**	**100**	99.5	**100**	99.7	91.9	**100**	99.6
Transistor	93.1	92.9	96.7	**100**	**100**	99.3	**100**	100
Zipper	**100**	**100**	98.5	98.8	99.9	99.7	**100**	**100**
Average.Object	97.4	98.5	98.0	99.1	99.5	98.2	**99.6**	**99.6**
Total.average	98.0	98.9	98.5	99.1	99.6	98.7	**99.7**	**99.7**

**Table 4:** Pixel-level AUROC (%) for various anomaly detection methods on MvTec AD. The best results are in bold, and the second-best results are underlined.

Method	DRAEM [46]	SSPCAB [47]	RD4AD [48]	PatchCore [25]	Simplenet [49]	CS-Flow [9]	MSFlow [10]	Ours
Carpet	95.5	95.0	98.9	99.1	98.2	95.2	**99.4**	**99.4**
Grid	**99.7**	99.5	99.3	98.7	98.8	94.4	99.4	99.4
Leather	98.6	99.5	99.4	99.3	99.2	89.3	**99.7**	**99.7**
Tile	99.2	**99.3**	95.6	95.9	97.0	90.7	98.2	98.0
Wood	96.4	**96.8**	95.3	95.1	94.5	91.2	97.1	96.2
Average.texture	97.9	98.0	97.7	97.6	97.5	92.2	**98.8**	98.5
Bottle	**99.1**	98.8	98.7	98.6	98.0	88.5	99.0	98.8
Cable	94.7	96.0	97.4	**98.5**	97.6	96.1	**98.5**	98.1
Capsule	94.3	93.1	98.7	98.9	98.9	98.1	99.1	**99.2**
Hazelnut	99.7	**99.8**	98.9	98.7	97.9	96.1	98.7	98.7
Metal_Nut	**99.5**	98.9	97.3	98.4	98.8	96.0	99.3	99.2
Pill	97.6	97.5	98.2	97.6	98.6	95.8	98.8	**99.1**
Screw	97.6	**99.8**	99.6	99.4	99.3	98.3	99.1	99.4
Toothbrush	98.1	98.1	**99.1**	98.7	98.5	97.4	98.5	98.4
Transistor	90.9	87.0	92.5	96.4	**97.6**	96.3	98.3	94.0
Zipper	98.8	99.0	98.2	98.9	98.9	95.8	99.2	99.1
Average.object	97.0	96.8	97.9	98.4	98.4	95.8	**98.8**	98.4
Total.average	97.3	97.2	97.8	98.1	98.1	94.6	**98.8**	98.4

**Table 5:** Image-level AUROC (%) and pixel-level AUROC (%) for various anomaly detection methods on BTAD. The best results are in bold.

Method	Patch-SVDD [23]	RealNet [21]	MSFlow [10]	Ours
Category 01	95.7/91.6	**100.0**/**98.2**	99.8/96.8	99.8/97.0
Category 02	72.1/93.6	88.6/96.3	91.4/97.4	**92.7**/**97.5**
Category 03	82.1/91.0	**99.6**/99.4	99.3/99.4	**99.6**/**99.5**
Average	83.3/92.1	96.1/97.9	96.8/97.8	**97.3**/**98.0**

**Table 6:** Results of different kernel sizes *K* on the VisA dataset. The best results are highlighted in bold.

	I-AUROC			P-AUROC		
K	3 × 3	5 × 5	7 × 7	3 × 3	5 × 5	7 × 7
candle	98.2	98.2	**98.7**	**99.4**	**99.4**	**99.4**
capsule	**97.2**	96.6	96.1	98.6	**99.7**	99.6
cashew	**98.6**	97.7	97.9	98.4	98.9	**99.1**
chewinggum	99.5	99.2	**99.6**	**99.4**	99.3	99.3
fryum	99.4	**99.8**	99.7	93.8	94.1	**94.2**
macaroni1	**98.4**	98.1	97.6	**99.8**	99.7	**99.8**
macaroni2	90.5	90.5	**90.7**	**99.6**	**99.6**	**99.6**
pcb1	**99.2**	99.1	98.7	**99.8**	**99.8**	**99.8**
pcb2	98.6	**98.9**	98.8	98.9	**99.3**	**99.3**
pcb3	98.1	**98.6**	**98.6**	99.3	99.3	**99.4**
pcb4	**99.8**	**99.8**	**99.8**	**99.0**	**99.0**	**99.0**
pipe_fryum	99.2	**99.6**	99.0	**99.2**	**99.2**	**99.2**
Average	**98.05**	98.00	97.93	98.76	98.94	**98.97**

**Table 7:** Results of different channel expansion factors α on the VisA dataset. The best results are highlighted in bold.

	I-AUROC				P-AUROC			
α	1	2	3	4	1	2	3	4
candle	**98.3**	**98.3**	98.2	98.2	**99.4**	**99.4**	99.4	99.4
capsule	95.8	**96.9**	96	96.6	99.6	99.6	**99.7**	**99.7**
cashew	97.8	98	**98.3**	97.7	**99.2**	98.8	98.9	98.9
chewinggum	**99.2**	**99.2**	**99.2**	**99.2**	99.3	**99.4**	**99.4**	99.3
fryum	98.6	**99.7**	99.2	**99.8**	93.7	**94.1**	93.5	**94.1**
macaroni1	98.2	97.1	**98.3**	98.1	**99.7**	**99.7**	**99.7**	**99.7**
macaroni2	88.9	89.2	89.8	**90.5**	99.5	**99.6**	**99.6**	**99.6**
pcb1	98.8	98.8	98.8	**99.1**	**99.8**	**99.8**	**99.8**	**99.8**
pcb2	98.7	98.3	98.6	**98.9**	99.2	98.9	99.2	**99.3**
pcb3	**98.6**	98.3	98.5	**98.6**	99.3	99.3	**99.4**	99.3
pcb4	**99.8**	**99.8**	**99.8**	**99.8**	**99**	**99**	**99**	**99.0**
pipe_fryum	99.2	**99.4**	**99.7**	99.6	99	99	99	**99.2**
Average	97.65	97.75	97.87	**98.00**	98.89	98.88	98.88	**98.94**

**Table 8:** Results of different channel attention mechanisms on the VisA dataset. The best results are highlighted in bold.

	I-AUROC			P-AUROC		
AM	SEBlock	ECA	Ours	SEBlock	ECA	Ours
candle	**98.4**	**98.4**	**98.4**	**99.4**	99.2	**99.4**
capusle	96.4	96.4	**96.8**	**99.7**	**99.7**	**99.7**
cashew	98.3	**98.4**	98.2	**98.8**	98.7	**98.8**
chewinggum	99.3	**99.6**	99.4	**99.4**	99.3	**99.4**
fryum	99.5	**99.7**	99.6	93.7	93.8	**94.5**
macaroni1	98.0	**98.5**	98.2	**99.7**	**99.8**	**99.8**
macaroni29	91.7	91.4	**92.3**	**99.6**	**99.6**	**99.6**
pcb1	98.8	98.8	**98.9**	**99.8**	**99.8**	**99.8**
pcb2	**98.8**	98.2	98.5	**99.2**	**99.2**	**99.2**
pcb3	98.7	98.8	**99.0**	**99.4**	**99.4**	**99.4**
pcb4	**99.8**	**99.8**	**99.8**	98.9	99	**99.1**
fryum	99.3	99.2	**99.5**	99.1	99.1	**99.2**
Average	98.07	98.10	**98.21**	98.88	98.89	**98.99**

## Data Availability

The datasets used in this study are all publicly accessible. MvTec AD is accessed through https://www.mvtec.com/company/research/datasets/mvtec-ad (accessed on 2 January 2025), VisA is accessed through https://amazon-visual-anomaly.s3.us-west-2.amazonaws.com/VisA_20220922.tar (accessed on 4 January 2025) and BTAD is accessed through https://avires.dimi.uniud.it/papers/btad/btad.zip (accessed on 14 May 2025).

## Abbreviations

The following abbreviations are used in this manuscript:

GCA	Global context-aware
AD	Anomaly detection
UAD	Unsupervised anomaly detection
H-Flow	Hierarchical flow
NLL	Negative log-likelihood
